# Inflammatory bowel disease phenotypes in diverse populations: a global comparative analysis

**DOI:** 10.1093/ecco-jcc/jjag051

**Published:** 2026-05-16

**Authors:** Charles N Bernstein, Gilaad G Kaplan, Zoann Nugent, Vineet Ahuja, Ashwin N Ananthakrishnan, Rupa Banerjee, Johan Burisch, Yan Chen, David Epstein, Angela J Forbes, Lorant Gonczi, Yubei Gu, Jeffri Gunawan, Dao Viet Hang, Jamilya Kaibullayeva, Uri Kopylov, Paolo G Kotze, Beatriz Iade, Peter L Lakatos, Julujak Limsrivilai, Jie Liang, Joyce W Y Mak, Siew Ng, Mirjam Severs, Jun Shen, Ajit Sood, Jesus K Yamamoto-Furusho, Shyla Yuan, Mads Damsgaard Wewer, Kaichun Wu, Jospeh W Windsor, Richard B Gearry, The Globalization Cluster of the International Organization for the Study of IBD

**Affiliations:** Department of Internal Medicine, University of Manitoba IBD Clinical and Research Centre, Max Rady College of Medicine, Rady Faculty of Health Sciences, University of Manitoba, Winnipeg, Manitoba, R3E3P4, Canada; Departments of Medicine and Community Health Sciences, University of Calgary, Calgary, Alberta, T2N4N1, Canada; Department of Internal Medicine, University of Manitoba IBD Clinical and Research Centre, Max Rady College of Medicine, Rady Faculty of Health Sciences, University of Manitoba, Winnipeg, Manitoba, R3E3P4, Canada; Department of Gastroenterology and Human Nutrition, All India Institute of Medical Sciences, New Delhi, 110029, India; Division of Gastroenterology, Massachusetts General Hospital, Boston, MA, 02114, United States; Department of Medicine, Harvard Medical School, Boston, MA, 02114, United States; IBD Center, Asian Institute of Gastroenterology, Hyderabad, 500032, India; Gastro Unit, Medical Section, Copenhagen University Hospital, Hvidovre, DK-2650, Denmark; Copenhagen Center for Inflammatory Bowel Disease in Children, Adolescents and Adults, Copenhagen University Hospital – Amager and Hvidovre, Hvidovre, DK-2650, Denmark; Department of Clinical Medicine, Faculty of Health and Medical Sciences, University of Copenhagen, Copenhagen, 2200, Denmark; Department of Gastroenterology, The Second Affiliated Hospital School of Medicine, Zhejiang University, Hangzhou, 310009, China; IBD Africa (NPO), Cape Town, South Africa with Department of Medicine, University of Stellenbosch, Western Cape, 7602, South Africa; Life Vincent Pallotti Hospital, Cape Town, Mowbray 7700, South Africa; Department of Medicine, University of Otago, Christchurch, 8011, New Zealand; Departement of Internal Medicina and Oncology, Semmelweis University Budapest, Budapest, H1085, Hungary; Department of Gastroenterology, Ruijin Hospital Affiliated to Shanghai Jiao Tong University School of Medicine, Shanghai, 200025, China; The JAG Centre for Inflammatory Bowel Disease Studies, Jakarta, 12160, Indonesia; Internal Medicine Faculty – Hanoi Medical University (HMU), Hanoi, 100000, Viet Nam; Research Institute of Cardiology and Internal Medicine, Almaty, 050000, Kazakhstan; IBD Unit, Sheba Medical Center, Gray School of Medicine, Tel Aviv University, Ramat Gan, 5262100, Israel; Health Sciences Postgraduate Program, Pontificia Universidade Católica do Paraná, Curitiba, 80440-220, Brazil; Association Crohn y Colitis Uruguay (ACCU), Montevideo, 11600, Uruguay; Department of Medicine, McGill University, Montreal, PQ, H3G2M1, Canada; Division of Gastroenterology, Siriraj Hospital Mahidol University, Bangkok, 10700, Thailand; Department of Gastroenterology, Xijing Hospital, Xi’an, 710032, China; Department of Medicine and Therapeutics, The Chinese University of Hong Kong, Shatin, New Territories, 999077, Hong Kong; Microbiota I-Center (MagIC), Department of Medicine and Therapeutics, Li Ka Shing Institute of Health Sciences, New Cornerstone Science Laboratory, The Chinese University of Hong Kong, Hong Kong SAR, 999077, China; Department of Gastroenterology and Hepatology, Radboudumc, Nijmegen, 6525 GA, the Netherlands; Department of Gastroenterology, Shanghai Renji, Hospital/Shanghai IBD Center, Shanghai, 200127, China; Department of Gastroenterology, Dayanand Medical College, Ludhiana, Punjab, 141001, India; Inflammatory Bowel Disease Clinic, Department of Gastroenterology, National Institute of Medical Sciences and Nutrition, Mexico City, 14080, Mexico; Division of Gastroenterology and Hepatology, Baoshan Branch, Renji Hospital, School of Medicine, Shanghai Jiao Tong University, Shanghai, 200444, China; Gastro Unit, Medical Section, Copenhagen University Hospital, Hvidovre, DK-2650, Denmark; Copenhagen Center for Inflammatory Bowel Disease in Children, Adolescents and Adults, Copenhagen University Hospital – Amager and Hvidovre, Hvidovre, DK-2650, Denmark; Department of Clinical Medicine, Faculty of Health and Medical Sciences, University of Copenhagen, Copenhagen, 2200, Denmark; Department of Gastroenterology, Xijing Hospital of Digestive Diseases, Fourth Military Medical University, Xi’an, 710032, China; Departments of Medicine and Community Health Sciences, University of Calgary, Calgary, Alberta, T2N4N1, Canada; Department of Medicine, University of Otago, Christchurch, 8011, New Zealand

**Keywords:** phenotype, global, cohorts, inflammatory bowel disease

## Abstract

**Background:**

The aim of this study was to determine if Crohn’s disease (CD) and ulcerative colitis (UC) manifest similarly in different parts of the world.

**Methods:**

Investigators of cohort studies from around the world were contacted to provide the most recent data from their CD and UC cohorts. Each cohort provided summated and averaged data on pre-specified variables. The Montreal Classification was used for phenotype assessment. Age at diagnosis was stratified into less than 17 years, 17-39 years, and >39 years. Disease location for CD was stratified as ileum only, colon only, ileocolon, upper gastrointestinal and proximal small bowel disease, and perineal penetrating disease. Disease behavior for CD was stratified into inflammatory disease, stricturing disease, and penetrating disease. Location for UC was stratified into proctitis, left-sided colitis, and extensive including subtotal and pancolitis. Reports were classified geographically as Asia, Latin America, South Africa, and “Western” (ie, North America, Europe, and Oceania). These categories were collapsed to “Western” and “non-Western” for analyses.

**Results:**

Data from 54 868 patients with IBD were included. For CD, data were available from nine Western cohorts, 12 Asian cohorts, three Latin American cohorts, and one African cohort. For UC, eight Western cohorts were compared with 11 Asian cohorts, three Latin cohorts, and one African cohort. The demographic and phenotypic distribution for CD and UC in comparisons between Western and non-Western countries were no different. Western cohorts had longer disease durations than non-Western cohorts

**Conclusion:**

No significant differences were seen in any phenotypic data between the cohorts, suggesting that IBD is similar worldwide.

## Graphical abstract

**Graphical Abstract:**
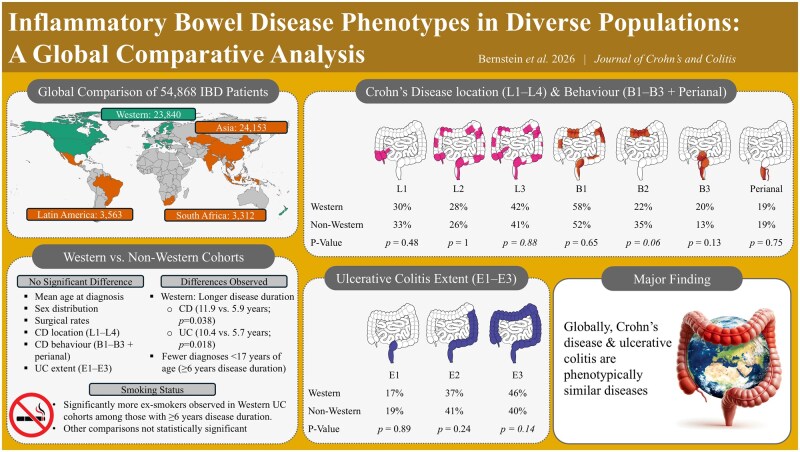

## Introduction

The causes of Crohn’s disease (CD) and ulcerative colitis (UC), the two main forms of inflammatory bowel disease (IBD), remain unknown. While a number of possible causes have been proposed—including dietary factors, ingestion of particulate matter with toxic additives, antibiotic use, climate change, and environmental antigens—many hypotheses involve changes to the gut microbiome.^1^^,^^2^ It is important to consider whether the emergence of IBD worldwide in jurisdictions with disparate diets, climates, and background genetics can lead to the identification of common causal factors that narrow down the etiologic search.^3^ While diets are quite different in China and Canada, for instance, might food additives be similar, or the impact of antibiotics on the gut microbiota be similar?^4^ A fundamental question that must be addressed to move the field forward is whether CD and UC manifest as the same diseases in different parts of the world. This will enable researchers to rationalize searching for common etiologies.

This question also holds practical importance. Modern clinical trials in IBD are extremely costly, and typically not repeated in each ethno-geographic population. Demonstrating that CD and UC are comparable across regions strengthens the rationale for generalizing trial results to broader populations. This will give regulatory bodies, clinicians, and patients confidence that results from clinical trials in one region may be valid in another. As individual clinical trials are often conducted in dozens of countries worldwide, confirming disease similarity in participating countries ensures that the results can be validly summated.

While the incidence and prevalence of IBD differs across the world, recent research has demonstrated similar patterns of disease emergence, albeit with different phases reflecting the temporal differences in disease emergence.^5^^,^^6^ However, clinical manifestations of disease presentation can be compared between regions to determine whether IBD is broadly similar around the world. With this goal in mind, members of the Global Cluster of the International Organization for the Study of IBD (IOIBD) collaborated with investigators worldwide to share registry data on the demographics and phenotype of CD and UC from their local institutions or countries.

## 2. Methods

Using an iterative process of contacting investigators of cohort studies from around the world, the most recent data from their CD and UC cohorts were collated. Each cohort provided ­summated and averaged data on pre-specified variables. The variables comprised demographic, clinical, and some treatment-specific data. The variables collected included: mean age at diagnosis; mean disease duration at most recent cohort update; gender; smoking status (current for CD and former for UC) at time of diagnosis; percentage who had undergone surgery for their CD or UC; disease location for CD and UC; and disease behavior for CD. The Montreal Classification was used for phenotype assessment.^7^ Age at diagnosis was stratified into less than 17 years, 17-39 years, and >39 years.^6^ Disease location for CD was stratified as L1 (ileum only), L2 (colon only), L3 (ileocolon), L4 (upper gastrointestinal and proximal small bowel disease), and p (perineal penetrating disease), according to the Montreal Classification. Disease behavior for CD was stratified into B1 (inflammatory disease), B2 (stricturing disease), and B3 (penetrating disease). The Montreal Classification was also used to stratify disease location for UC into E1 (proctitis), E2 (left-sided colitis), and E3 (extensive including subtotal and pancolitis). The most recent Montreal Classification of phenotype was used for all registries (rather than the phenotype at incidence), and the entire history of IBD-related surgeries was included where available.

## 3. Analysis

The data reports were transferred to an Excel™ spreadsheet and subsequently read by SAS™ v.9.4 for analysis. Three age groups were considered in keeping with the Montreal Phenotypic Classification: <17 years, 17-39 years, and 40 years and older. Reports were originally classified geographically as Asia, Latin America, South Africa, and “Western” (ie, North America, Europe, and Oceania). These categories were collapsed to “West” and “Other” for analyses carried out using the Wilcoxon two-sample test. A *P*-value of <.05 was considered significant for differences. Missing data varied across cohorts; therefore, each cohort was included in analyses for which relevant data were available. When a variable was not collected in a given jurisdiction (eg, smoking), that cohort was excluded from that specific analysis but retained in all other analyses. Three distinct comparative analyses were undertaken. First, outcomes were compared between Western and non-Western cohorts including all patients regardless of disease duration. Second, Western and non-Western cohorts were compared among individuals with at least 6 years’ disease duration. Third, outcomes were compared between multicenter cohorts and single-center cohorts.

## 4. Results

Data from 54 868 patients with IBD were included in the analysis. From Latin America there were data on 3563 patients, from South Africa there were 3312, from Asia there were data from 24 153 patients and from western regions there were data from 23 840 patients. For CD, data were available from nine Western cohorts, which included: Canada (14 centers), USA (one center), European regions (22 centers) as well as national data from each of Denmark, Holland, Hungary, and Switzerland (one center each), and Israel (one center), and New Zealand (one center). Twelve Asian cohorts including China (Chinese Database for IBD [CHASE], which is a cohort of seven centers, plus three other centers), Hong Kong (one center), India (three regional centers), Indonesia (three centers), Kazakhstan (one center), Thailand (one center), and Vietnam (one center). Also, there were three Latin American cohorts including Brazil (one center), Mexico (one center), and Uruguay (one center), and one African cohort from South Africa. In total, nine Western cohorts were compared with 16 non-Western centers (Table 1, refs 8–30)

**Table 1.** Number of participants from each jurisdiction.

Country	Cohort make-up		Subjects		Cohort years of accrual
			CD	UC	
**Western**			9	7	
**Canada^8^ ^,^ ^9^**	14 centers, national, prevalent		2112	1865	2017-2023
**USA^10^**	1 center, referral-based, prevalent		1793	1300	2005-2023
**Denmark^11^ ^,^ ^12^**	national incident		213	300	2003-2011
**Europe^13^ ^,^ ^14^**	22 centers, incident		548	873	2010-2020
**Holland^15^**	8 centers, national, prevalent		2118	1269	2007-2015
**Hungary^16^**	5 centers, national incident		357	378	2009-2018
**Israel^17^**	1 center, referral-based, prevalent		3535	1669	2010-2023
**New Zealand^18^**	1 center, national, prevalent		2323	1587	2018-2023
**Switzerland^19^**	1 center, national, prevalent		1600		2006-2015
**Total**			14 599	9241	
**Asia**	Cohorts		12	11	
**China^20^**	7 centers, national, incident, prevalent		1354	1081	2000-2024
**China^21^ ^,^ ^22^**	1 center, prevalent		1173	621	2019-2024
**China^a^**	1 center, prevalent		1257	522	2016-2024
**China^a^**	1 center, prevalent		939		2010-2021
**Hong Kong^23^**	4 centers, prevalent		131	135	2011-2018
**India^a^**	1 regional center, incident, prevalent		412	1676	2021-2024
**India^24^ ^,^ ^25^**	1 regional center, incident, prevalent		1611	4531	2001-2023
**India^26^**	1 regional center, incident, prevalent		3367	3711	2010-2024
**Indonesia^27^**	3 centers, incident		55	23	2021-2024
**Kazakhstan^a^**	1 center, incident, prevalent		331	775	2018-2023
**Thailand^a^**	1 referral center, incident, prevalent		181	190	2018-2023
**Vietnam^a^**	6 centers (3 cities), incident, prevalent		30	47	2022-2023
**Total**			10 841	13 312	
**Latin America**	Cohorts		3	3	
**Brazil^28^**	1 referral center, prevalent		322	178	2015-2021
**Mexico^29^**	42 centers, incident, prevalent		501	2072	2000-2017
**Uruguay^30^**	28 centers, incident, prevalent		159	331	2006-2023
**Total**			982	2581	
**South Africa^a^**	1 center, incident, prevalent		1478	1834	2003-2024
**Total non-Western**		13 301		17 727	

a No publication available for this cohort.

For UC eight western cohorts were included with data from Canada (14 centers collated), USA (one center), Europe (22 centers collated), Denmark (national), Holland (eight centers), Hungary (national), Israel (one center), and New Zealand (one center). Eleven Asian cohorts included China (CHASE Cohort of seven centers, plus two other centers), Hong Kong (one center), India (three regional centers), Indonesia (three centers), Kazakhstan (one center), Thailand (one center), and Vietnam (one center). There were three Latin cohorts including Brazil (one center), Mexico (one center), and Uruguay (one center), and one African cohort from South Africa. In total, eight Western cohorts were compared to 15 non-Western centers (Table 1).

Table 2 shows that median age at diagnosis, sex distribution, and surgical rates were generally similar between Western and non-Western cohorts for both CD and UC, with no statistically significant differences. Smoking patterns were arithmetically different between Western and non-Western countries but the differences were not statistically different. However, Western cohorts had significantly longer disease duration for both CD (median 11.9 vs 5.9 years, *P* = .038) and UC (10.4 vs 5.7 years, *P* = .018). Phenotypic characteristics, including disease location and behavior in CD and extent of disease in UC, were comparable between regions.

**Table 2.** Demographics and phenotype data for CD and UC from Western cohorts compared to all non-Western cohorts.

Variables	Crohn’s disease			Ulcerative colitis		
	Western cohorts	Non-Western cohorts	*P*-value	Western cohorts	Non-Western cohorts	*P*-value
**Data sources**	9	16		8	15	
**Median age at diagnosis, years**	33	34	.50	35	37	.38
**Minimum, maximum**	26, 43	28, 45		31, 42	31, 52	
**Diagnosed prior to age 17, %**	11	6	.13	8	3	.054
**Diagnosed age 17-39, %**	62	63	1	62	55	.11
**Diagnosed age 40+, %**	25	31	.31	29	40	.13
**Median disease duration, years**	**11.9**	**5.9**	**.038**	**10.4**	**5.7**	**.018**
**Minimum, maximum, years**	**7.7, 15.6**	**1.5, 15.5**		**7.3, 14.7**	**1.1, 10.4**	
**Percentage female**	52	40	.066	51	44	.27
**Percentage with surgery**	29	27	.17	8	4	.23
**Percentage smokers**	31	10	.081	9	6	.24
**Percentage ex-smokers at diagnosis**				27	16	.067
**Crohn’s disease, %**						
**L1**	29	32	.93			
**L2**	29	23	.24			
**L3**	36	45	.20			
**L4**	6	3	.25			
**B1**	57	53	.47			
**B2**	21	29	.11			
**B3**	19	12	.17			
**Perineal disease**	18	25	.58			
**Ulcerative colitis, %**						
**E1**				17	21	.43
**E2**				38	37	.97
**E3**				43	38	.30

The bolder entries are those that are statistically significant.

### 4.1. Comparison of Western and non-Western centers with disease duration of at least 6 years

In this comparison data were not available from Holland, Switzerland, and one of the Chinese cohorts, but all other centers provided data limited to individuals with at least 6 years’ disease duration. Significantly fewer individuals were diagnosed before age 17 in non-Western compared with Western countries (for CD 7% vs 12%, *P* = .036 and for UC 3% vs 8%, *P* = .02). Western countries also reported a higher proportion of ex-smokers in UC (30% vs 15%, *P* = .03). Otherwise, demographic characteristics and disease phenotype were similar between Western and non-Western cohorts (Table 3).

**Table 3.** Demographics and phenotype data for CD and UC from Western cohorts compared to all non-Western cohorts including only those with at least 6 years’ disease duration.

Variables	Crohn’s disease			Ulcerative colitis		
	West	Non-West	*P*-value	West	Non-West	*P*-value
**Data sources**	7	15		7	14	
**Median age at diagnosis, years**	30	34	.081	34	38	.13
**Minimum, maximum**	24, 38	27, 51		28, 41	31, 57	
**Diagnosed prior to age 17, %**	**12**	**7**	**.026**	**8**	**3**	**.021**
**Diagnosed age 17-39, percent**	62	61	.82	57	57	.92
**Diagnosed age 40+, %**	24	33		38	40	
**Median disease duration, years**	13	11	.28	14	11	.44
**Minimum, maximum, years**	9, 20	8, 19		8, 20	6, 19	
**Percentage female**	48	44	.37	52	52	1
**Percentage with surgery**	31	36	.90	10	6	.35
**Percentage smokers**	24	11	.29	10	6	.52
**Percentage ex-smokers at diagnosis**	22	4	.10	**30**	**15**	**.033**
**Crohn’s disease, %**						
**L1**	29	31	.83			
**L2**	24	28	.73			
**L3**	45	40				
**L4**						
**B1**	55	54	.97			
**B2**	25	28	.32			
**B3**	22	13				
**Perineal disease**	20	17	.92			
**Ulcerative colitis, %**						
**E1**				15	19	.74
**E2**				38	40	.26
**E3**				46	38	

The bolder entries are those that are statistically significant.

### 4.2. Comparison of outcomes from multi-center cohorts versus single center cohorts

Median disease duration was longer for UC subjects reported by single centers (14 vs 9 years, *P* = .04). Otherwise, there were no differences in demographic or phenotype outcomes when comparison was of data from multi-center versus single-center cohorts (Table 4).

**Table 4.** Demographics and phenotype data for CD and UC from multicenter cohorts compared to single center cohorts.

Variables	Crohn’s disease			Ulcerative colitis		
	Multi-center	Single-center	*P*-value	Multi-center	Single-center	*P*-value
**Data sources**	7	15		7	14	
**Median age at diagnosis, years**	34	31	.89	38	37	.28
**Minimum, maximum**	26, 42	24, 51		31, 57	28, 48	
**Diagnosed prior to age 17, %**	9	8	.94	2	5	.36
**Diagnosed age 17-39, %**	62	61	.82	60	57	.44
**Diagnosed age 40+, %**	27	31		40	39	
**Median disease duration, years**	10	13	.084	**9**	**14**	**.039**
**Minimum, maximum, years**	8, 20	9, 19		6, 18	9, 20	
**Percentage female**	53	47	.23	47	52	.74
**Percentage with surgery**	27	39	.24	6	9	.35
**Percentage smokers**	28	11	.17	10	6	.70
**Percentage ex-smokers at diagnosis**	17	11	.69	23	20	.31
**Crohn’s disease, %**						
**L1**	24	35	.054			
**L2**	36	25	.19			
**L3**	47	40				
**L4**						
**B1**	55	54	.97			
**B2**	25	28	.32			
**B3**	22	13				
**Perineal disease**	12	23	.13			
**Ulcerative colitis, %**						
**E1**				17	16	.69
**E2**				38	40	.74
**E3**				44	40	

The bolder entries are those that are statistically significant.

## 5. Discussion

We compared CD cohorts from 25 centers in 18 countries along with a multi-country report from Europe, and UC cohorts from 22 centers in 16 countries plus a multi-country report from Europe, to determine similarity of these diseases across diverse jurisdictions worldwide. For both CD and UC, age at diagnosis, sex distribution, disease location, and disease behavior for CD were broadly similar between Western and non-Western centers. Disease duration at the time of reporting the cohorts was significantly longer for Western cohorts, reflecting the more recent emergence of IBD in non-Western regions, and the earlier initiation of data collection in Western countries. When the analyses were restricted to individuals with at least 6 years’ disease duration, the only differences between Western and non-Western cohorts were significantly fewer individuals with CD or UC diagnosed prior to age 17, differences in smoking cessation in UC, and differences in disease duration between single- and multi-center reports. Otherwise, comparisons of Western versus non-Western cohorts with disease duration of at least 6 years and a comparison of multicenter cohorts versus single-center cohorts yielded similar results. Overall, CD and UC presented with similar demographic and phenotypic characteristics worldwide.

Smoking patterns seemed to differ between regions as 31% of individuals with CD from Western cohorts were smokers compared to 10% in non-Western cohorts. For UC, 27% were ex-smokers in Western countries versus 16% in non-Western countries. While not statistically significant, these findings suggest that smoking has a greater impact on IBD in Western populations. While smoking has been reported to adversely affect the disease course in Western populations with CD,^31^ Indian studies from different jurisdictions showed no impact of smoking on CD risk^32^ and no impact of smoking on disease course.^33^ Thus, smoking may have differential effects depending on regional differences in background genetics, environment, and/or the composition of the intestinal microbiome.

Overall, this large international comparison of IBD cohorts from across the world provides evidence that CD and UC are phenotypically similar diseases regardless of where they manifest in the world. This provides some foundation to pursue common etiologies worldwide either individually or through large collaborative efforts. The evolving environments across the world, as developing countries become more westernized, provide opportunities to determine drivers of disease etiology through comparative studies such as those in the ACCESS cohort and the subsequent GIVES-21 study.^34^

Clinical studies that recruit from diverse IBD populations worldwide generally demonstrate comparable disease phenotypes. While genetic differences across jurisdictions worldwide may shape immune responses, and geography and dietary variations may influence the gut microbiome, disease distribution in the gastrointestinal tract in CD and UC, as well as disease behavior in CD, seems consistent across the world. As the global burden of IBD continues to rise, recognizing and addressing modifiable environmental determinants offers a critical opportunity to implement standardized preventative health strategies aimed at reducing incidence and mitigating long-term disease burden.^35^

The median disease duration for the Western cohorts was 10–12 years and while there may be some progression to more complex disease over time, much of the disease evolution has probably occurred. However, with a median of only 6 years there yet may be evolution to more stricturing and penetrating disease in non-Western cohorts. However, when analyses were restricted to individuals from contributing jurisdictions with at least 6 years’ disease duration, thereby excluding those early in their disease course, phenotypes were similar worldwide. That the phenotypes are similar despite different disease duration between Western and non-Western countries might suggest that for the majority of individuals their ultimate phenotype is declared early in their disease course. Future research is necessary to determine whether IBD responds similarly to therapy worldwide, Given the substantial differences in genes, environmental exposures, and gut microbiomes worldwide between populations, identifying shared commonalities across diverse populations will be critical to advancing our understanding of IBD pathogenesis on a global scale.^36^

This study has a number of strengths. To our knowledge, this is the largest study of its type to compare IBD cohorts from around the world with more than 50 000 individuals with IBD divided between Asia, Latin America, South Africa, and Western Countries compared with regard to demographic, phenotypic, and clinical variables. Comparing western and non-western cohorts has enabled sub-phenotypes to be compared with significant power to detect differences in a wide range of variables. The use of established cohorts that have been well-described and the majority of which have already been published makes the comparison particularly robust. Finally, the use of established phenotypic criteria with hard end-points also reduces error in comparison between the groups.

This study also has some weaknesses that need to be considered when interpreting the data.^37^ First, the extent to which the cohorts in the study reflect IBD within each country is uncertain. Many countries were not represented, and the included cohorts varied in design, with some being population-based and others regional center-based. Several centers are located in regions with low IBD prevalence; however, these cohorts are particularly valuable as they represent IBD in geographically diverse areas of the world.

Cohorts derived from referral center registries may not reflect the full disease burden within a country, and for large countries such as China and India, patient data were not available from many jurisdictions. However, the study still included a substantial number of individuals with IBD from both China and India. Even though many were accrued from major urban centers, this represents an important starting point and provides one of the few opportunities to compare IBD phenotypes across many of the countries included. Further, data accrual was not uniform across countries. Until more consistent national approaches to accessing patients are developed, studies will probably continue to rely on convenience samples derived from IBD registries at referral centers in many jurisdictions.

Additionally, disease phenotype changes with time. The present study did not specifically measure change in phenotype (eg, inflammatory to stricturing behavior) over time, which may be another important means of determining whether there are differences between centers. However, despite the duration of follow up being longer in the westernized countries, no significant differences were seen in any other phenotypic data between the cohorts. Analyzing data from all centers with affected persons with at least 6 years’ disease duration only confirmed consistent similarities between disease phenotypes. Finally, some regions of the world were less well represented than others. This is particularly true of Africa where there were fewer established cohorts. However, through our networks at IOIBD, WGO, and WHO, we were able to proactively identify sites from a diverse set of countries. We acknowledge that these limitations may reduce the generalizability of our findings.

In a recent comprehensive expert review on goals for optimizing the research agenda and clinical management of the burgeoning burden of IBD worldwide, it was suggested that there remain significant gaps in the timely diagnosis and appropriate management in many countries.^38^ The authors called for improved healthcare policies in countries where IBD is just emerging, improved access to care, and enhanced patient support systems. It would strengthen the lobbying of governments to enhance IBD care if it could be shown that the diseases getting advanced care and support in developed countries are the same diseases in emerging countries. Showing that CD and UC are similar diseases worldwide, as we have done in our phenotype comparison study, provides evidence that CD and UC require similar management worldwide. The authors of this expert review issued a call for standardizing epidemiological data accrual that would require standardized phenotyping across jurisdictions. They reported that standardization would “enable more effective comparisons of IBD across different regions, helping to build cascading guidelines that reflect both global insights and local healthcare resource limitations.” Hence, our international IBD phenotype comparison is timely and meeting an important research mission proposed by a body of international IBD experts. Defining the burden of IBD does not simply mean how many individuals have the disease, but also what clinical burden of the disease and, hence, documenting disease phenotype also defines the disease burden.

In conclusion, this large international comparison demonstrates that the clinical phenotype of CD and UC is remarkably consistent across diverse geographic regions, despite differences in disease duration, environmental exposures, and genetics. These findings support the feasibility of global collaborative research efforts in IBD and suggest that core disease characteristics may be driven by shared mechanisms. Further studies are needed with prospective data collection and increased numbers of centers that represent non-Western regions, especially from Africa and Latin America.

## Supplementary Material

jjag051_Supplementary_Data

## Data Availability

The datasets presented in this article are not readily available. All available data are presented herein.
